# Reversible Half Wave Rectifier Based on 2D InSe/GeSe Heterostructure with Near‐Broken Band Alignment

**DOI:** 10.1002/advs.201903252

**Published:** 2021-01-04

**Authors:** Yong Yan, Shasha Li, Juan Du, Huai Yang, Xiaoting Wang, Xiaohui Song, Lixia Li, Xueping Li, Congxin Xia, Yufang Liu, Jingbo Li, Zhongming Wei

**Affiliations:** ^1^ Henan Key Laboratory of Photovoltaic Materials, School of Physics Henan Normal University Xinxiang 453007 China; ^2^ State Key Laboratory of Superlattices and Microstructures, Institute of Semiconductors, Chinese Academy of Sciences & Center of Materials Science and Optoelectronics Engineering University of Chinese Academy of Sciences Beijing 100083 China; ^3^ State Key Laboratory for Artificial Microstructures and Mesoscopic Physics, School of Physics Peking University Beijing 100871 China; ^4^ Henan Key Laboratory of Infrared Materials & Spectrum Measures and Applications Henan Normal University Xinxiang 453007 China; ^5^ Institute of Semiconductors South China Normal University Guangzhou 510631 China

**Keywords:** 2D van der Waals heterostructures, gate‐tunable reversible rectifiers, GeSe, InSe, near broken band alignment

## Abstract

2D van der Waals heterostructures (vdWHs) offer tremendous opportunities in designing multifunctional electronic devices. Due to the ultrathin nature of 2D materials, the gate‐induced change in charge density makes amplitude control possible, creating a new programmable unilateral rectifier. The study of 2D vdWHs‐based reversible unilateral rectifier is lacking, although it can give rise to a new degree of freedom for modulating the output state. Here, a InSe/GeSe vdWH‐FET is constructed as a gate‐controllable half wave rectifier. The device exhibits stepless adjustment from forward to backward rectifying performance, leading to multiple operation states of output level. Near‐broken band alignment in the InSe/GeSe vdWH‐FET is a crucial feature for high‐performance reversible rectifier, which is shown to have backward and forward rectification ratio of 1:38 and 963:1, respectively. Being further explored as a new bridge rectifier, the InSe/GeSe device has great potential in future gate‐controllable alternating current/direct current convertor. These results indicate that 2D vdWHs with near‐broken band alignment can offer a pathway to simplify the commutating circuit and regulating speed circuit.

## Graphical abstract

A gate‐induced reversible AC/DC convertor is realized based on InSe/GeSe heterojunction with near‐broken band alignment. The device exhibits backward and forward rectification ratio of 1:38 and 963:1, respectively. A bridge rectifier based on the InSe/GeSe vdWHs is also proposed to simplify the commutating circuit and regulating speed circuit.

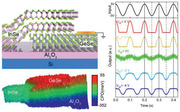

## 1 Introduction

A rectifier is a kind of electronic device which can convert alternating current (AC) to direct current (DC) by using one or more pn junction diode made up of two semiconducting materials.^[^ ^1^ ^]^ In the pn junction diode, spontaneous charge transfer across the interface, due to the difference of the Fermi levels of these two semiconductors, creates a potential barrier hindering the electron current transferred from n‐type side to p‐type side, leading to rectifying effect. The Fermi levels of conventional semiconductors are often determined by dopants. It is well known that we can achieve Zener diode, Esaki diode and IMPATT (IMPact ionization Avalanche Transit Time) diode by controlling the doping level in n‐ or p‐type side. However, if a diode is produced, it will be used in a certain well‐established circuit to realize one specific design purpose. Especially, conventional diodes with certain doping concentration can only demonstrate a fixed unidirectional conduction. With the advent of the Internet of things (IoT) era, however, multifuctional electronic device is becoming more important to satisfy the demands of the diversified application and circuit simplification.^[^ ^2^ ^]^

Gate‐controlled logic rectifier based on van der Waals heterostructures (vdWHs), vertically stacked with two or more 2D semiconductors,^[^ ^3^, ^4^, ^5^ ^]^ has received tremendous attentions because of their potential applications in tunable rectifying circuit.^[^ ^6^, ^7^ ^]^ For example, with the help of the ambipolar property, the black phosphorous (BP)/WSe_2_^[^ ^8^ ^]^ and BP/graphene^[^ ^9^ ^]^ vdWHs exhibit gate‐tunable logic rectification. In addition, the asymmetric vertically stacked MoTe_2_/MoS_2_ vdWH also demonstrates high tunable rectifying property.^[^ ^10^ ^]^ The presented results in MoTe_2_/MoS_2_ vdWH indicate that the logic rectifying property can be induced by the charge‐carrier transport switching between tunneling and thermal activation. Besides, the Au/hBN/MoS_2_ tunneling vdWHs also show a gate‐controlled switch from pn to np diode, paving the way for future logic rectifier.^[^ ^11^ ^]^ However, the tunneling current is sensitive to the thickness of hBN, leading to great obstruction in practical applications. Thus, we investigate the vdWHs‐based logic rectifier in this work.

**Figure 1:**
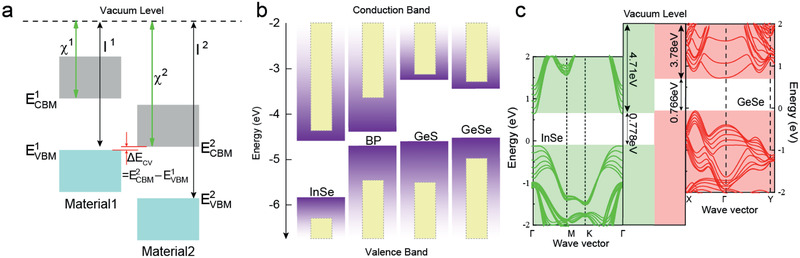
a) Schematic illustration of absolute band positions in the type‐II band alignment with respect to the vacuum level. b) Band alignments obtained from VASP of different 2D materials (bulk and monolayer) are indicated by filled violet gradient column and yellow solid column, respectively. Data are extracted from the ref. ^[^ ^54^, ^55^, ^56^ ^]^. c) Band structures of InSe and GeSe with their electron affinities and bandgaps obtained from our DFT calculation using PBE method, suggesting near broken‐gap band alignment in the heterostructure.

In theory, most vdWHs with type‐II band alignment are very useful for pn diodes with forward rectifying mode,^[^ ^4^ ^]^ since they possess large band offsets on one side (either conduction or valence band). The ultrathin nature of 2D semiconductors can contribute to the Fermi level shift as a function of the gate bias, even resulting in the rectifying‐mode conversion between backward and forward rectification.^[^ ^7^ ^]^ According to the band alignment, backward rectification originates from the incipient band‐to‐band tunneling (BTBT) effect,^[^ ^10^ ^]^ whereas, forward rectification results from the normal ideal majority carrier recombination current.^[^ ^12^ ^]^ Consequently, reversible logic rectifier should still be based on a type‐II heterostructure with an easy activation of BTBT effect. From the schematic diagram illustrated in **Figure** **1**a, we note that the initial difference between the valence band maximum (VBM) of Material1 and the conduction band minimum (CBM) of Material2, relating to the ionization potential (*I*) and electron affinity (*χ*) of each material system, is an important aspect of electronic properties for the type‐II heterostructure, that is $\Delta E_{\mathrm{CV}}\;=E_{\mathrm{CBM}}^{2}\;-\;E_{\mathrm{VBM}}^{1}=I^{1}-\chi^{2}$. When Δ*E*_CV_ is in the range of 0–0.2 eV, this highly staggered type‐II band alignment is called near‐broken band alignment. It could be an ideal situation for easy inversion by applying a moderate negative bias voltage, turning on BTBT current through the ultrathin vdW gap.^[^ ^13^ ^]^

Recently, 2D heterostructures with near‐broken band alignment have aroused great interests. It has been found that the BP with moderate and tunable ionization potential provides an ideal platform for constructing broken or near broken heterostructure, such as BP/SnSe_2_,^[^ ^14^ ^]^ BP/MoS_2_^[^ ^12^ ^]^ and BP/ReS_2_.^[^ ^15^ ^]^ Hu and co‐workers developed a heterostructure tunneling device consisting of black arsenic phosphorus (b‐AsP) and indium selenide (InSe), which shows a record high reverse rectification ratio exceeding 10^7^.^[^ ^16^ ^]^ However, these devices always exhibit a single backward rectifying mode due to the small bandgap (≈0.3 eV) of Group‐V atomic crystals, leading the formation of isotype (nn) heterojunctions under a certain external gate bias.^[^ ^16^, ^17^ ^]^ Moreover, 2D group‐VA materials (P or As) are unstable in air and tend to degrade very rapidly in ambient air, which restricts them further applications.^[^ ^18^ ^]^ After searching in the 2D atomic semiconductor library, WSe_2_ is identified as a promising candidate for the p‐type terminal.^[^ ^19^, ^20^ ^]^ Li et al.^[^ ^21^ ^]^ have reported an ultrahigh reverse rectifying behavior of vertically stacked WSe_2_/SnSe_2_ device. However, the WSe_2_/SnS_2_^[^ ^22^ ^]^ and WSe_2_/InSe^[^ ^23^ ^]^ vdWHs demonstrate a high forward rectifying ratio. Fan et al. ascribed this contradiction to the different doping level of WSe_2_.^[^ ^24^ ^]^ The relatively low hole concentration of the intrinsic WSe_2_ hinders the BTBT current under negative drain bias, leading a typical forward rectifying behavior. After 2% V doping, a backward rectifying diode is manifested. Consequently, most vdWHs exhibit single rectifying behavior and the gate electrical field can only modulate the magnitude of rectification ratio, limiting logic rectifier performance and integration. Novel 2D vdWHs with near‐broken band alignment need to be explored for logic rectifier.

Germanium selenide (GeSe), whose structure bears a strong resemblance to that of BP, has attracted considerable attention recently due to its air‐stability, fascinating in‐plane anisotropic properties and fast photoresponse.^[^ ^25^ ^]^ Furthermore, according to the previous studies, GeSe process a small work function (WF) of 4.4–4.8^[^ ^26^, ^27^ ^]^ approaching to the electron affinity of InSe (4.6 eV),^[^ ^28^, ^29^ ^]^ as shown in Figure 1b. In GeSe single crystals, the acceptor level was located at ≈37.8 meV above valance band maximum and the hole carrier concentration is ≈5.31 × 10^16^ cm^−3^ at room temperature.^[^ ^30^ ^]^ These features predict that bulk or multilayer‐ InSe/GeSe vdWH‐FET is an interesting alternative combination with the near‐broken‐gap configuration. Here, we successfully constructed a vdWH based on multilayer InSe and GeSe. Theoretical and experimental techniques are used to investigate the band alignment and the results show that the InSe/GeSe vdWHs have a near‐broken band alignment. The device can function as a backward diode and forward rectifying diode by applying negative and positive gate voltage, respectively, leading to multiple operation states of output level. Furthermore, we have proposed a polarity‐switchable and magnitude‐controllable rectifier based on the InSe/GeSe vdWHs, which show great potential in gate‐tunable logic electronic application.

## 2 Results and Discussion

We first studied the band alignment of the InSe/GeSe vdWHs by first‐principles calculations based on density functional theory (DFT) in Figure 1c. The slab models containing six layers InSe or GeSe are used in DFT calculation, respectively, due to the fact that CBM and VBM of InSe or GeSe could only change within the range of 1–5 layers, that is, if the thicknesses of these flakes are >5 layers, no obvious variation in CBM and VBM positions is expected.^[^ ^26^, ^31^ ^]^ The reference level is adopted as the energy plateau in this vacuum region of the plane‐averaged electrostatic potential for the isolated InSe and GeSe crystals (Figure S1, Supporting Information). Figure 1c demonstrates the band offset between InSe and GeSe forces a near‐broken band alignment. In Figure S2 (Supporting Information), we also computed the band structure of bulk InSe and GeSe, and the accurate band edges are obtained using the Kraut's method^[^ ^32^ ^]^ with respect to the Se 3s core level. The result is in accordance with that of the vacuum level alignment method. Consequently, the InSe/GeSe vdWH has an intrinsic near‐broken band alignment, allowing us to use it as a model system.

**Figure 2:**
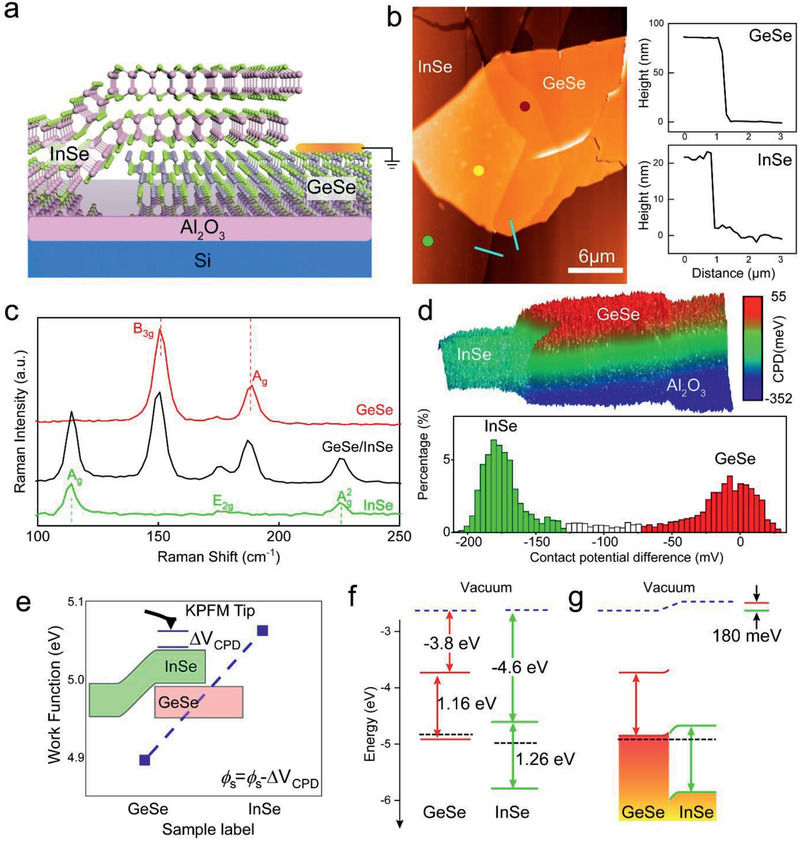
a) Schematic illustration of the InSe/GeSe heterostructure b) AFM image of the InSe/GeSe vdWH‐FET sample and the thicknesses of the GeSe (top) and the InSe flakes (bottom) corresponding to the blue lines. c) Raman spectra of the InSe/GeSe vdWH‐FET (yellow), isolated InSe (green) and GeSe layers (red). d) 3D KPFM mapping image of the InSe/GeSe vdWH (top) and histogram distributions of Δ*V*_CPD_ extracted from the KPFM mapping image (bottom). e) Work function values of InSe and GeSe. f,g) The band alignment of the InSe/GeSe vdWH before and after contact.

To experimentally verify the band alignment of InSe/GeSe, we performed Kelvin probe force microscopy (KPFM) measurements. **Figure** **2**a presents schematic diagrams of KPFM measurement on an Al_2_O_3_/Si substrate. An InSe flake was transferred onto a mechanically cleaved GeSe flake by a dry‐transfer method (the optical image of the InSe/GeSe vdWH can be found in Figure S3 (Supporting Information) and experimental details are provided in Figure S4 (Supporting Information)). The surface morphology of the junction area is clean and smooth without obvious air bubbles. The thickness of InSe and GeSe flakes (Figure 2b) are about 20 and 80 nm, respectively. The quality of the GeSe, InSe, and heterostructure was confirmed by Raman spectra depicted in Figure 2c. Raman spectrum taken from the red dot in Figure 2b exhibits two peaks at around 150 and 188 cm^−1^ for the GeSe nanoflake, corresponding to the B_3g_ mode (out‐of‐plane opposite vibration of Ge and Se atoms in zigzag direction) and A_g_ mode (in‐plane opposite vibration of Ge and Se atoms in armchair directions).^[^ ^33^, ^34^ ^]^ In the Raman spectrum collected from the green dot, one can see three distinct peaks located at 116 cm^−1^(A_g_), 177 cm^−1^ (E_2g_), and 226 cm^−1^($A_{g}^{2}$), which are all induced from the first‐order scattering from optical phonons of InSe flakes.^[^ ^35^ ^]^ Comparing the Raman spectra of the InSe/GeSe vdWHs with the individual GeSe or InSe layers, one can note that the vibrational modes of GeSe (B_3g_, A_g_) soften by ≈1.0–1.6 cm^−1^, while those of InSe harden by ≈0.8–1.0 cm^−1^. Such minor shift in these Raman modes indicates the vdW interlayer coupling between bottom GeSe and top InSe layers.

Figure 2d shows the corresponding in situ KPFM mapping images of the InSe/GeSe vdWH. The KPFM measurements were performed using platinum/iridium (Pt/Ir)‐coated Si tip. Before measuring, the tip was calibrated on a highly oriented pyrolytic graphite (HOPG) surface, whose WF is well known to be 4.6 eV (Figure S5, Supporting Information). From KPFM results, the WF value of the GeSe can be estimated to be about 4.88 eV (Figure 2e), coinciding with result reported in previous literature.^[^ ^27^ ^]^ While, the larger WF value (≈5.06 eV) of InSe flakes, compared with the InSe flakes with low‐density of surface states (≈4.8 eV),^[^ ^28^ ^]^ may be due to the surface oxidation in the air.^[^ ^36^ ^]^ The surface potential difference (SPD) of the InSe/GeSe vdWH‐FET is determined to be about 180 mV. Based on the obtained DFT and KPFM results and the previously reported band properties (CBM, VBM, and *E*_g_) of GeSe and InSe, we graphically described the predicted energy band alignment of the InSe/GeSe vdWH at equilibrium before and after contact (Figure 2f,g). Here, the electron affinity and bandgap of InSe (GeSe) are around 4.6 (3.8) and 1.26 (1.16) eV based on the previously reported band properties,^[^ ^25^, ^28^, ^29^, ^37^ ^]^ respectively. Micro‐photoluminescence (µPL) spectroscopy is characterized to confirm the band alignment and the results are depicted in Figure S6 (Supporting Information). The PL quenching ratio (PL Intensity InSe /PL Intensity InSe/GeSe) is calculated to be 145 times, which implies that the charge separation occurs in the heterostructure region due to the large difference in WF.^[^ ^38^, ^39^, ^40^ ^]^

**Figure 3:**
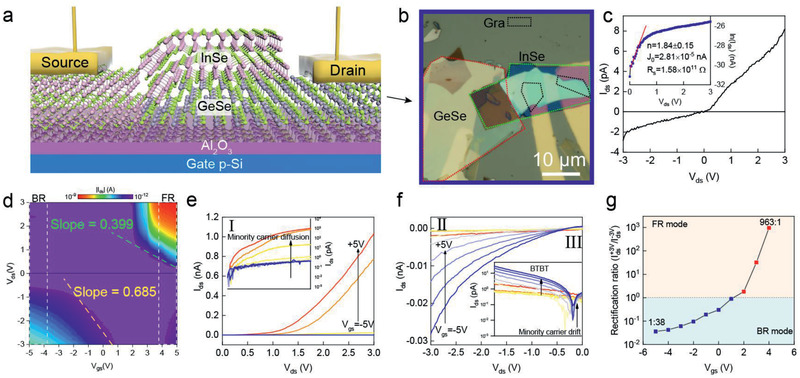
Schematic diagram a) and optical microscope image b) of a complete InSe/GeSe vdWH‐FET device. c) *I*_ds_–*V*_ds_ characteristics of the InSe/GeSe device at *V*_g_ = 0 V in linear and logarithmic (inset) scales. d) Color map of output curves (*I*_ds_ vs *V*_ds_) at different gate voltages. e,f) Gate‐tunable output curves (*I*_ds_–*V*_ds_) of the device under positive and negative drain bias in linear and logarithmic (inset) scales, respectively. g) The rectification ratio of the vdW diode at different gate voltage.

**Figure** **3** shows the electrical properties of the vertically stacked multilayer InSe/GeSe vdWH on a 30 nm Al_2_O_3_/Si substrate. The thicknesses of InSe and GeSe are 32.6 and 68.3 nm, respectively, which are close to that of the KPFM sample. GeSe serves as the drain terminal, whereas InSe is grounded and serves as the source terminal. To avoid the potential resist residue in conventional lithography techniques, Au electrodes were transferred onto the vdWH by a photolithographic‐pattern‐transfer (PPT) method.^[^ ^41^ ^]^ Few‐layer graphene (Gr) was intercalated into the Au/InSe interface to modify the Schottky barrier for Gr‐InSe contacts due to its tunable work function.^[^ ^42^ ^]^ The current–voltage (*I*–*V*) curve in Figure 3c clearly exhibits a typical pn junction behavior at room temperature. The diode coefficient *n* of ≈1.84 was calculated by fitting the *I*–*V* curve in forward bias range (more detail in Note S1, Supporting Information). This value indicates that the forward‐bias current is dominated by interlayer recombination, similar to the common p–n vertical vdWHs. In addition, the reverse current starts to increase dramatically at a critical reverse voltage of about −2.8 V, signifying a Zener diode characteristic. In traditional silicon device, if a breakdown voltage is lower than 4*E*_g_/*q*, the breakdown mechanism can be assigned to Zener tunneling.^[^ ^43^, ^44^, ^45^ ^]^ Here, *E*_g_ is the bandgap of the semiconductor, and *q* is the charge of an electron. Considering the bandgap of GeSe (*E*_g_ = 1.16 eV) and InSe (*E*_g_ = 1.26 eV), the value of 4*E*_g_/*q* is 4.4 V. Therefore, such a room‐temperature reverse breakdown can be attributed to Zener breakdown originating from BTBT. In short, the current transport over the InSe/GeSe vdWH displays as a composite of forward interlayer recombination and reverse tunneling mechanisms.

In Figure 3d, the conversion of *I*–*V* curves at different gate voltage shows an approximate asymmetric *V*_ds_ polarization. The color map indicates a transition from forward rectifying mode (FR mode, region I) to backward rectifying mode (BR mode, region II) modulated by gate voltage, instead of the rather symmetric *V*_ds_ polarization with ON state. Surprisingly, when *V*_ds_ potential is higher than 0.3 V, the device exhibits typical n‐type characteristic with an on/off ratio of ≈10^3^, however, when *V*_ds_ potential is lower than −0.8 V, the device starts to exhibits typical p‐type with an on/off ratio of ≈10^1^. Figure S7 (Supporting Information) shows the polar‐switchable field effect curve in detail and the comparison of the on/off ratio of the InSe/GeSe vdWH and the previous reported systems. Moreover, according to the slopes of drain‐to‐gate voltage gain (*V*_ds_ vs *V*_gs_) in Figure 3d, the gate efficiency was calculated to be 0.399 and 0.685 for positive and negative *V*_ds_ region, respectively. It is indicative of the formation of a stronger coupling coefficient between Fermi level and the electrostatic gate under negative bias; that is, BTBT current is more sensitive to the electrostatic gate than interlayer recombination current. Line cuts at fixed *V*_gs_ are shown in Figure 3e,f. Figure 3g shows the forward‐to‐reverse drain current ratio at the fixed gate *V*_ds_ = ±3 V extracted from e and f. The device exhibits backward and forward rectification ratio of 1:38 and 963:1 under gate bias of ±5 V, respectively. Two operation modes can be clearly identified, and the transition occurs at *V*_gs_ ≈ +1 V. Comparing with the state‐of‐art vdWH‐FETs, such as MoS_2_/WSe_2_,^[^ ^46^ ^]^ BP/MoS_2_,^[^ ^12^ ^]^ WSe_2_/SnS_2_,^[^ ^19^ ^]^ AsP/MoS_2_^[^ ^17^ ^]^ and so on, the InSe/GeSe vdWH‐FET realizes the reversible rectifying effect. Figure S8 (Supporting Information) displays the electrical properties in detail. For *V*_gs_ = +5 V, the diode exhibits a normal forward rectifying characteristic. However, as the *V*_gs_ decreases to −5 V, the reverse current shows a dramatic increase at −0.5 V, the forward current is suppressed approaching 1 pA. The gate leakage current curves at fixed *V*_gs_, in Figure S8 (Supporting Information), show independent of drain‐voltage with a value of <5 pA. This can rule out any influence of the gate leakage to the rectifying transition. We also fabricated the InSe/GeSe vdWH‐FET on a 285 nm SiO_2_/Si substrate (Figure S9, Supporting Information). The device also demonstrated reversible rectifying characteristic. For comparison, the rectifying performance of the different devices is summarized in **Tables** **1** and **2**. The devices on Al_2_O_3_/Si substrate demonstrates better rectification ratio and higher gate efficiency. This enhancement comes from the high *k* value (6.4) of Al_2_O_3_, which can hold more charge in the channel and decrease physical gate thickness in FET. When the heavily doped Si substrate with 300 nm SiO_2_ dielectric layer is used as the back gate, the gate capacitance is relatively small, which means the electrostatic gate control of the channel per unit gate voltage is not strong. Furthermore, our results also show more flexible operability of the InSe/GeSe vdWH than that of other vdWHs in previous reports due to the unique near‐broken band alignment.

**Table 1:** Summary of the rectifying performance of forward rectification ratio (FRR), back rectification ratio (BRR) and gate efficiency (GE) of the transistors based on our InSe/GeSe vdWHs on the Al_2_O_3_/Si substrate, SiO_2_/Si substrate, where rectification ratio is defined as $I_{\mathrm{ds}}^{\mathrm{positive}\;V}$ /$I_{\mathrm{ds}}^{\mathrm{negative}\;V}$

Devices on different substrates	*k*‐value of dielectric	FRR	BRR	GE
Al_2_O_3_/Si	6.4	≈1 × 10^3^	≈2 × 10^−2^	0.399/0.685
SiO_2_/Si	3.9	≈5 × 10^1^	≈2 × 10^−1^	0.028/0.072

**Table 2:** Comparison of the rectifying performance between the InSe/GeSe vdWH and the previous reported systems, including forward rectification ratio (FRR), back rectification ratio (BRR), and gate efficiency (GE)

Parameters	InSe/GeSe	ReSe_2_/MoS_2_^[^ ^57^ ^]^	AsP/InSe^[^ ^16^ ^]^	WSe_2_/SnSe_2_^[^ ^21^ ^]^
Reversible rectifying	Yes	No	No	No
GE	0.399/0.685	–	–	–
FRR	≈1 × 10^3^	≈5 × 10^2^	–	–
BRR	≈2 × 10^−2^	–	10^−7^ ^a)^	10^−8^ ^a)^

^a)^ In the refs. ^[^ ^15^ ^]^ and ^[^ ^22^ ^]^ the backward rectification ratio was defined as $I_{\mathrm{ds}}^{\mathrm{negative}\;V}$ /$I_{\mathrm{ds}}^{\mathrm{positive}\;V}$. Different calculation method leads to the reciprocal results.

To understand the obtained electrical behavior of the InSe/GeSe vdWH‐FET, the electrical transport of both the individual GeSe and InSe FET were separately measured and the results are shown in Figures S10 and S11 (Supporting Information). The linear *I*–*V* characteristics of the GeSe FET indicate Ohmic contact between Au and GeSe in consistent with previous reports.^[^ ^47^ ^]^ The corresponding transfer curve exhibits a strong p‐type transport behavior, which means the Fermi level is close to the VBM of GeSe and its hole concentration is high.^[^ ^48^ ^]^ We also investigated the performance of the Gr‐GeSe‐Gr device in Figure S11 (Supporting Information). The nonlinear transport feature suggests a Schottky barrier between Gr and GeSe nanoflakes. Additionally, in Figure S12 (Supporting Information), the nonlinear *I*_ds_–*V*_ds_ curves of the Gr‐InSe‐Gr device obviously indicate the large Schottky barrier at the present Gr‐InSe interface. In contrast, according to the Schottky–Mott rule, the electron Schottky barrier height is nearly zero when graphene and InSe are contacted each other ideally,^[^ ^49^ ^]^ and an early indication of the Ohmic contact was observed in the in‐planar devices.^[^ ^28^, ^31^ ^]^ This discrepancy may arise from the surface defects^[^ ^28^ ^]^ introduced through the transfer process because InSe is sensitive to moisture, and oxidation occurs at the surface as exposed in the air.^[^ ^50^ ^]^ Combined with the KPFM results, the Fermi level of InSe is pinned at 5.06 eV, which may be associated with defect states such as substituted structure of Se by O atom.^[^ ^51^ ^]^ From the transfer curves of the Gr‐InSe‐Gr device, a positive gate voltage to attain the turn‐on state seems to corroborate the presence of the Fermi lever pinning effect. We also carried out the transport measurement on the few‐layer Gr as function of *V*_g_. In Figure S13 (Supporting Information), the hole doping feature of the graphene can be deduced from the positive sign of the charge neutral gate voltage. Thus, the charge transport mechanism across the Gr‐InSe interface combines thermoelectron emission and tunneling processes, as shown in the inset of Figure S12 (Supporting Information). At a large positive *V*_gs_, the electron concentration in InSe increases dramatically and the Fermi level of few‐layer Gr approaches the Dirac cone, reducing the tunneling barrier height. Thus, the tunneling effect governs the charge transport in the Gr‐InSe device. Based on these results, we can conclude that a p^+^‐n‐G heterojunction is formed in the Gr/InSe/GeSe vdW devices at large positive gate voltage.

The device working mechanism can be explained in detail based on the energy band diagrams at different bias conditions as shown in Figure S14 (Supporting Information). At equilibrium state, when GeSe and InSe contact with each other, electrons will move into InSe from GeSe, leaving more holes in GeSe, thus creating a type‐II band alignment with large band offset. Additionally, the Schottky barrier formed at the Gr‐InSe interface due to the surface oxidation is also considered. At positive drain bias, in Figure S14b (Supporting Information), the total current is dominated by the interlayer recombination current (*J*_Re_), which is common in vdWHs due to strong Coulomb interaction. Due to the high hole density and the Ohmic contact with Au, the hole injection from Au electrode to GeSe exhibits a very weak gate modulation. At the same time, for *V*_gs_ > 1 V, the electron concentration in InSe increases dramatically and more electrons can tunnel through the surface oxide layer, increasing forward recombination current. However, for *V*_gs_ < 1 V, the electron injection from graphene to InSe is suppressed by the energy barrier related to the surface oxidation layer. When a negative drain bias voltage is applied to the GeSe side, the external electric field counteracts the build‐in field. Therefore, VBM of GeSe moves above CBM of InSe forming a type‐III band alignment at the InSe/GeSe interface. The Schottky barrier height at Gr‐InSe interface is also reduced. Thus, the electron transport mechanism is mainly dominated by the BTBT effect with the help of the external field. The negative electrostatic gate voltage results in the narrow tunneling barrier, further increasing the tunneling probability. However, when the large positive *V*_gs_ is applied, the GeSe channel comes into the depletion state and the width of the depletion region increases. Therefore, a pn junction is formed at the InSe/GeSe interface, resulting in a low reverse saturation current and a high break down voltage exceeding the measure range. In general, the near‐broken band alignment in the InSe/GeSe vdWH is a crucial feature for the reversible rectification ratio. Meanwhile, the Schottky barrier at the Gr‐InSe interface further reduces the forward current at negative gate bias, leading a backward rectifying behavior. Surprisingly, a transition state in the reverse bias was also observed in Figure 3f (region III). We attribute this phenomenon to the minority carrier drift current. Since, under small negative *V*_ds_, the conduction and valence bands on opposite sides of the vdWH are not enough close to occur BTBT effects, the electric field sweeps the mobile electrons in GeSe portion into InSe portion. With a positive voltage applied to the backgate terminal, more electrons will be accumulated in GeSe channel, inducing a relatively higher reverse current.

**Figure 4:**
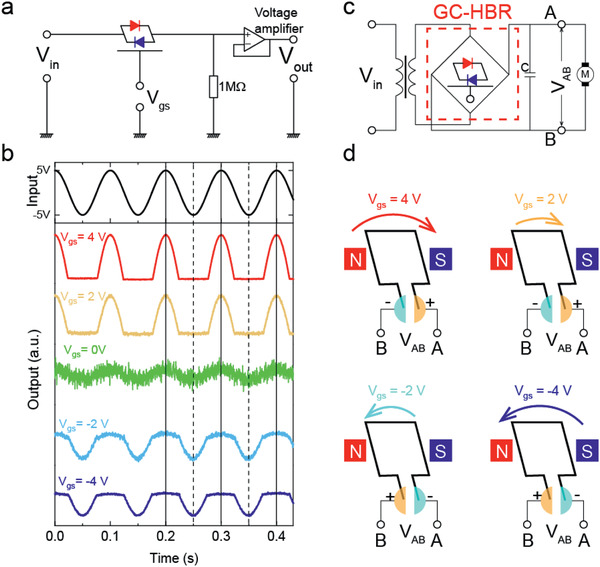
Gate‐tunable rectification of an analog harmonic signal in the InSe/GeSe vdWH‐FET. a) The schematic of the polarity switchable half‐wave rectifier. b) The half‐wave rectifying results with input wave amplitude of 5 V (harmonic signal ≈10 Hz) under gate voltage of −4 to 4 V, respectively. c,d) The proposed gate‐controlled heterojunction bridge rectifier (GC‐HBR) and its future application scenarios.

With the unique gate‐tunable rectifying behavior, the InSe/GeSe vdWHs can open up unique opportunities in analog circuit applications. **Figure** **4** shows the performance of the gate‐tunable half wave rectifier circuit based on the InSe/GeSe vdWH. When a cosinusoidal wave is input in the InSe/GeSe FET, output wave starts from a positively rectified half wave in the largest electron doping side, and can be tuned into a negatively rectified half wave in the hole doping side. Obviously, the negatively rectified half wave is very limited, thus cannot meet the requirement for practical application. It might be attributed to the low mobility of GeSe (Figure S10 and Note S2, Supporting Information), severely hindering the electrons transporting from the source to the drain electrodes. Since more and more novel 2D materials are emerging, we believe the improvement of the reverse rectifying property is possible.

We then proposed an AC/DC conversion concept called the gate‐controlled heterojunction bridge rectifier (GC‐HBR), as illustrated in Figure 4c. The GC‐HBR is analogous to the conventional bridge rectifier except that vdWH‐FETs are used instead of pn junction diodes. Four vdWH‐FETs are integrated on the same one substrate and wired through electrical connections. As a result, circuit efficiency of GC‐HBR is higher than the gate‐tunable half wave rectifier. Gate voltage can simultaneously apportion reversed action over all four vdWH‐FETs. The tunable rectifying characteristic of near‐broken band alignment vdWH‐FET should endow the GC‐HBR with adjustable magnitude and steerable polarity, which represents a possible practical multifunctional bridge rectifier with important potential as a new form of AC/DC convertor. We connect the GC‐HBR with filter capacitor and DC motor to realize the speed and direction control. This system is proposed to simplify the primary components in typical AC/DC pulse width modulation (PWM) convertors and offers a specific platform to realize 2D vdWHs‐based integrated electronics.

## 3 Conclusion

In summary, this work realizes the gate‐controlled reversible rectifier based on an artificially stacked van der Waals heterostructure of GeSe and InSe. The band alignment of the InSe/GeSe vdWHs, investigated by theoretical and experimental techniques, exhibits near‐broken band alignment. By positive gate voltage modulation of the band alignment at heterostructure interface, the significant reverse tunneling current is suppressed in such thin p–n junctions. In contrast, with negative gate voltage, the near broken band alignment favors the BTBT effect at negative drain bias, and the forward recombination current is hindered by reducing the electron injection from electrodes to InSe portion. Consequently, the device exhibits fully reversible forward to backward rectifying performance, leading to multiple operation states of output level. More importantly, by taking advantage of the near‐broken band alignment, the polarity‐switchable behavior can be explored for creating gate‐tunable rectifier. The results pave the way for the implementation of InSe/GeSe vdWH‐FET as a promising candidate for future multifunctional AC/DC convertor.

## 4 Experimental Section

##### Synthesis of Single Crystals

Single crystal InSe and GeSe was synthesized by the Bridgman method. In, Ge, and Se powders were purchased from Pioneer Materials Inc. with 99.999% metal basis. Equimolar mixture of In and Se powder was loaded into a quartz tube, which was evacuated and sealed by oxy‐hydrogen flame. Then the sealed tube was heated to 685 °C with 70 min and then heated to 700 °C within 5 min and kept at 700 °C for 3 days. After that, it was cooled to 500 °C within 2 days followed by naturally cooling. GeSe single crystals were synthesized at the same experimental conditions by using stoichiometric amounts of raw materials.

##### Preparation of vdW Heterostructure

Multilayer GeSe was exfoliated from bulk crystal using Nitto tape and directly transferred onto a highly p‐doped silicon substrates covered by 30 nm Al_2_O_3_. High‐*k* Al_2_O_3_ dielectric was deposited on Si wafer by atomic layer deposition (ALD). InSe was micromechanically exfoliated onto a separate PDMS film on a glass slide and transferred in contact with a GeSe flake under the optical microscope assisted by an aligned transfer system. The thickness of GeSe flakes (≈80 nm) and InSe (≈20 nm) was initially identified by an optical microscope and finally determined by an atomic force microscope. Few‐layer graphene was micromechanically exfoliated and transferred onto the surface of InSe nanosheets to reduce the Schottky barrier. The Raman spectra were obtained using a LabRAM‐HR Evolution Raman spectrometer with an excitation wavelength of 532 nm. Subsequently, ≈40 nm Au electrodes were transferred onto heterostructure diode by using a photolithographic‐pattern‐transfer (PPT) method.^[^ ^41^ ^]^ The constructed device was finally obtained after being immersed in acetone to remove the remaining PMMA, and then annealed at 200 °C for 1 h in a 10% H_2_/Ar environment to remove the residual organic pollution, resulting in a good contact and high‐quality interface. All the direct‐current electrical characterizations were performed using a Keysight B1500A digital source meter.

##### Simulation Methods

The first principles calculations based on density functional theory as implemented in the Vienna Ab initio simulation package. Moreover, the Perdew–Burke–Ernzerhofer (PBE) method is adopted to describe the electronic properties and the projected augmented wave (PAW) potential is also employed to model the electron‐ion potential.^[^ ^52^ ^]^ Although the bandgap values are underestimated by PBE, the band offset trend is still worth taking as a reference.^[^ ^53^ ^]^ The kinetic energy cutoff is set as 500 eV for the plane wave expansion. In addition, the convergence criterions of energy and forces are chosen as 10^−5^ eV and 0.01 eV Å^−1^, respectively.

## Conflict of Interest

The authors declare no conflict of interest.

## Supporting information

Supporting Information
