# Supplementary material for: Reversible Half Wave Rectifier Based on 2D InSe/GeSe Heterostructure with Near‐Broken Band Alignment
Source: Adv Sci (Weinh). 2021 Jan 4;8(4):1903252. doi: 10.1002/advs.201903252 (PMC7887575; doi:10.1002/advs.201903252)
Supplement: Supplementary file 1 — Supporting Information [file ADVS-8-1903252-s001.pdf]

© 2020 Wiley-VCH GmbH

ADVANCED SCIENCE
Open Access

Supporting Information

for *Adv. Sci.,* DOI: 10.1002/advs.201903252

Reversible Half Wave Rectifier Based on 2D InSe/GeSe Heterostructure with Near-Broken Band Alignment

Yong Yan, Shasha Li, Juan Du, Huai Yang, Xiaoting Wang, Xiaohui Song, Lixia Li, Xueping Li, Congxin Xia, Yufang Liu,* Jingbo Li,* and Zhongming Wei**

Supporting Information

Reversible Half Wave Rectifier Based on 2D InSe/GeSe Heterostructure with Near-Broken Band Alignment

Yong Yan, Shasha Li, Juan Du, Huai Yang, Xiaoting Wang, Xiaohui Song, Lixia Li, Xueping Li, Congxin Xia, Yufang Liu,* Jingbo Li,* and Zhongming Wei**

Dr. Y. Yan, S. Li, J. Du, Dr. X. Song, Dr. X. Li, Prof. C. Xia
Henan Key Laboratory of Photovoltaic Materials
School of Physics
Henan Normal University
Xinxiang 453007, China
E-mail: xiacongxin@htu.edu.cn

H. Yang, Dr. X. Wang, Prof. Z. Wei
State Key Laboratory of Superlattices and Microstructures
Institute of Semiconductors
Chinese Academy of Sciences & Center of Materials Science and Optoelectronics Engineering
University of Chinese Academy of Sciences
Beijing 100083, China
Email: zmwei@semi.ac.cn

Dr. L. Li, Prof. Y. Liu
Henan Key Laboratory of Infrared Materials & Spectrum Measures and Applications
Henan Normal University
Xinxiang 453007, China
Email: yf-liu@htu.edu.cn

Prof. J. Li
Institute of Semiconductors, South China Normal University
Guangzhou 510631, China
Email: jbli@m.scnu.edu.cn

J. Du
State Key Laboratory for Artificial Microstructures and Mesoscopic Physics
School of Physics
Peking University
Beijing 100871, China

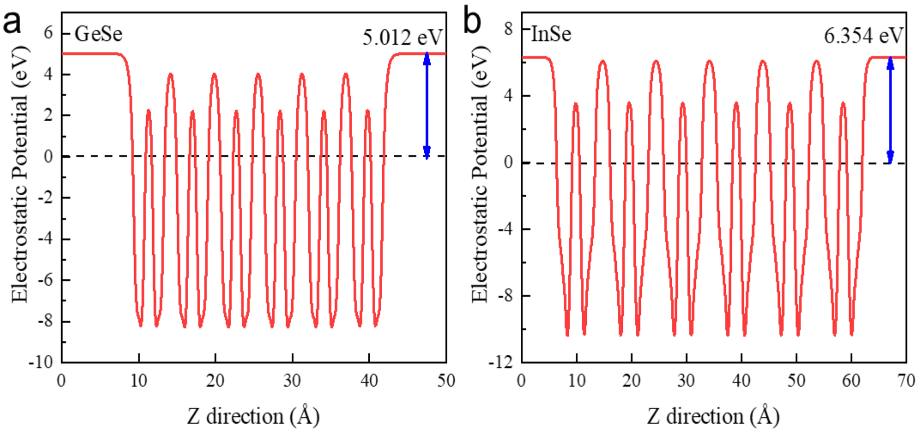

Figure S1 Electrostatic potential of GeSe (a) and InSe (b), respectively.

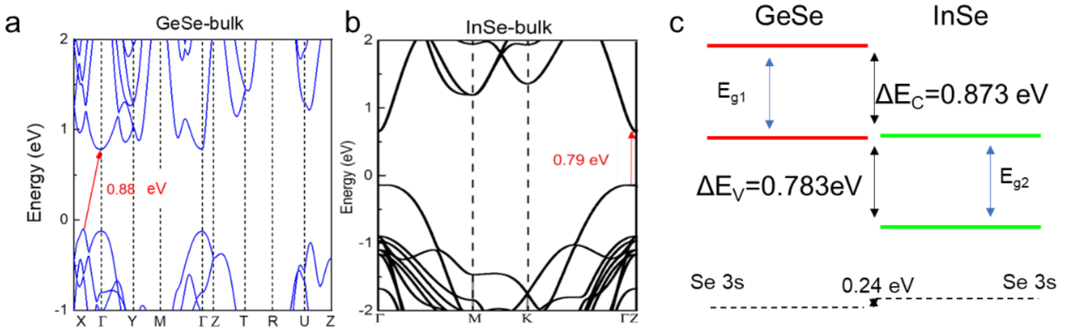

Figure S2 (a, b) Calculated band structures of bulk InSe and GeSe using PBE method based on the first principles calculations. (c) Calculated band edges of bulk InSe and GeSe with respect to the Se 3s core level.

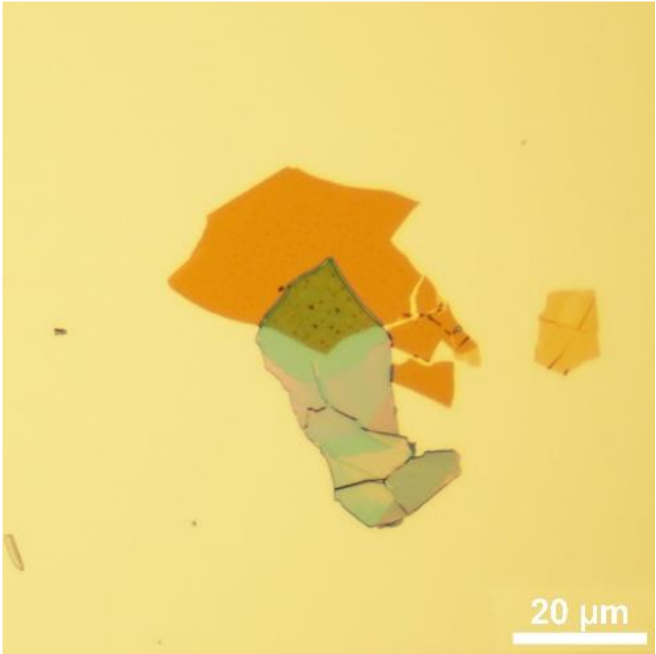

Figure S3 Optical images of the InSe/GeSe vdWH fabricated by mechanically transferring the InSe flake onto the GeSe flake.

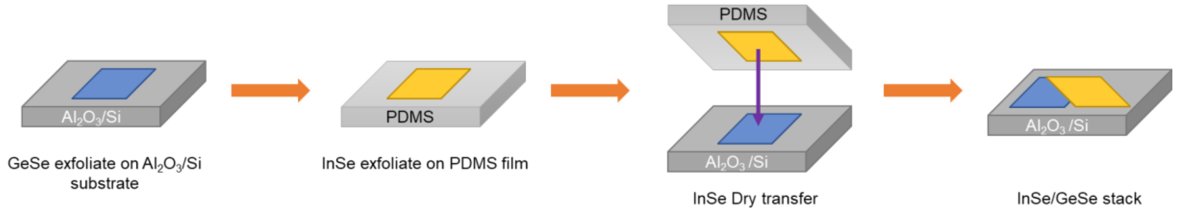

Figure S4 Schematic fabrication procedure of the InSe/GeSe vdWH on Al_2O_3/Si substrates.

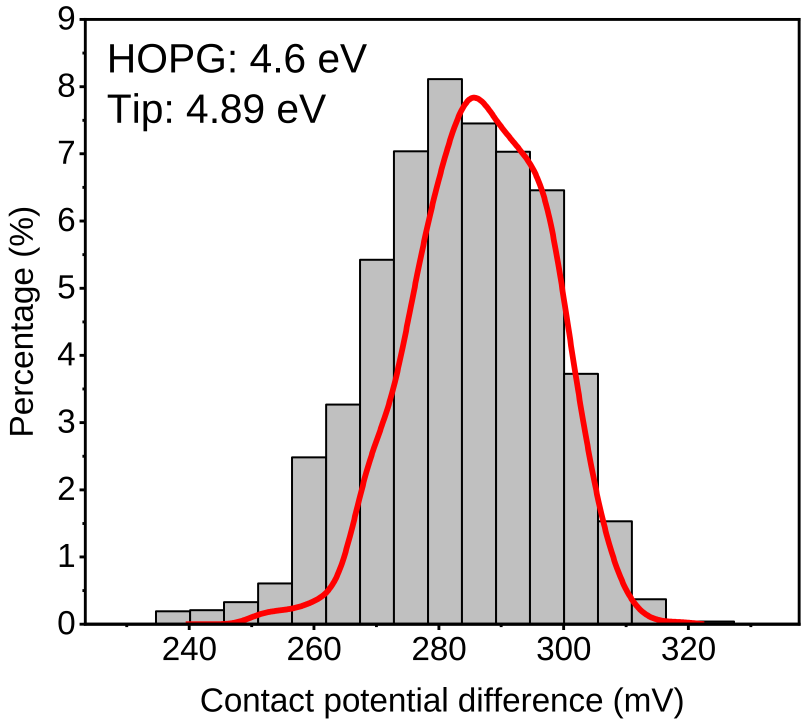

Figure S5 Histogram of contact potential difference of the HOPG surface.

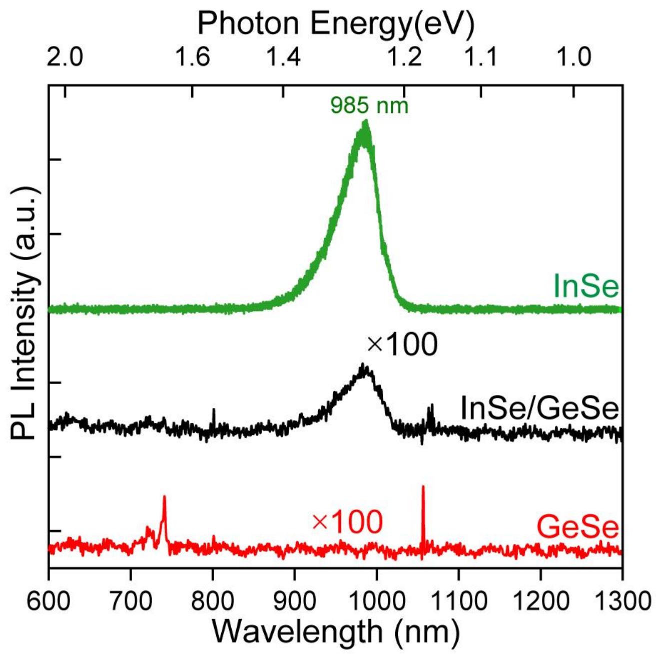

Figure S6 μPL spectra observed from the InSe/GeSe vdWH (Yellow), isolated InSe (green) and GeSe layers (red) in Figure 2b.

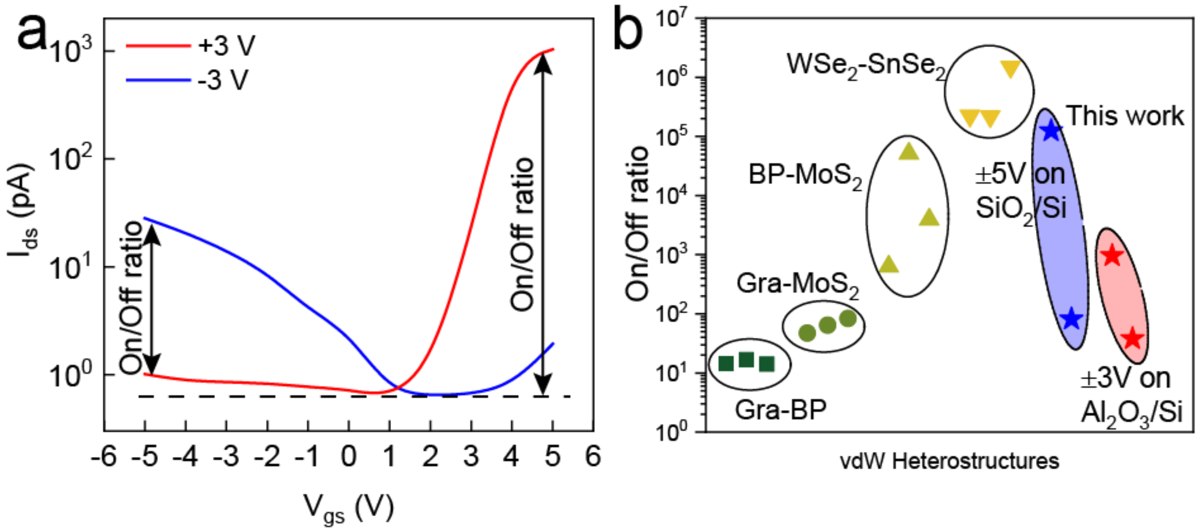

Figure S7 (a) I_{ds}-V_{gs} transfer characteristics of the InSe/GeSe device on Al_2O_3/Si substrate in logarithmic scale under different drain bias. (b) Current on/off ratio comparison of different vdW heterostructure devices. Reference data from the works based on WSe_2-$SnSe_2$ [1], MoS_2/BP [2], Graphene/MoS_2 [3], and Graphene/BP [4].

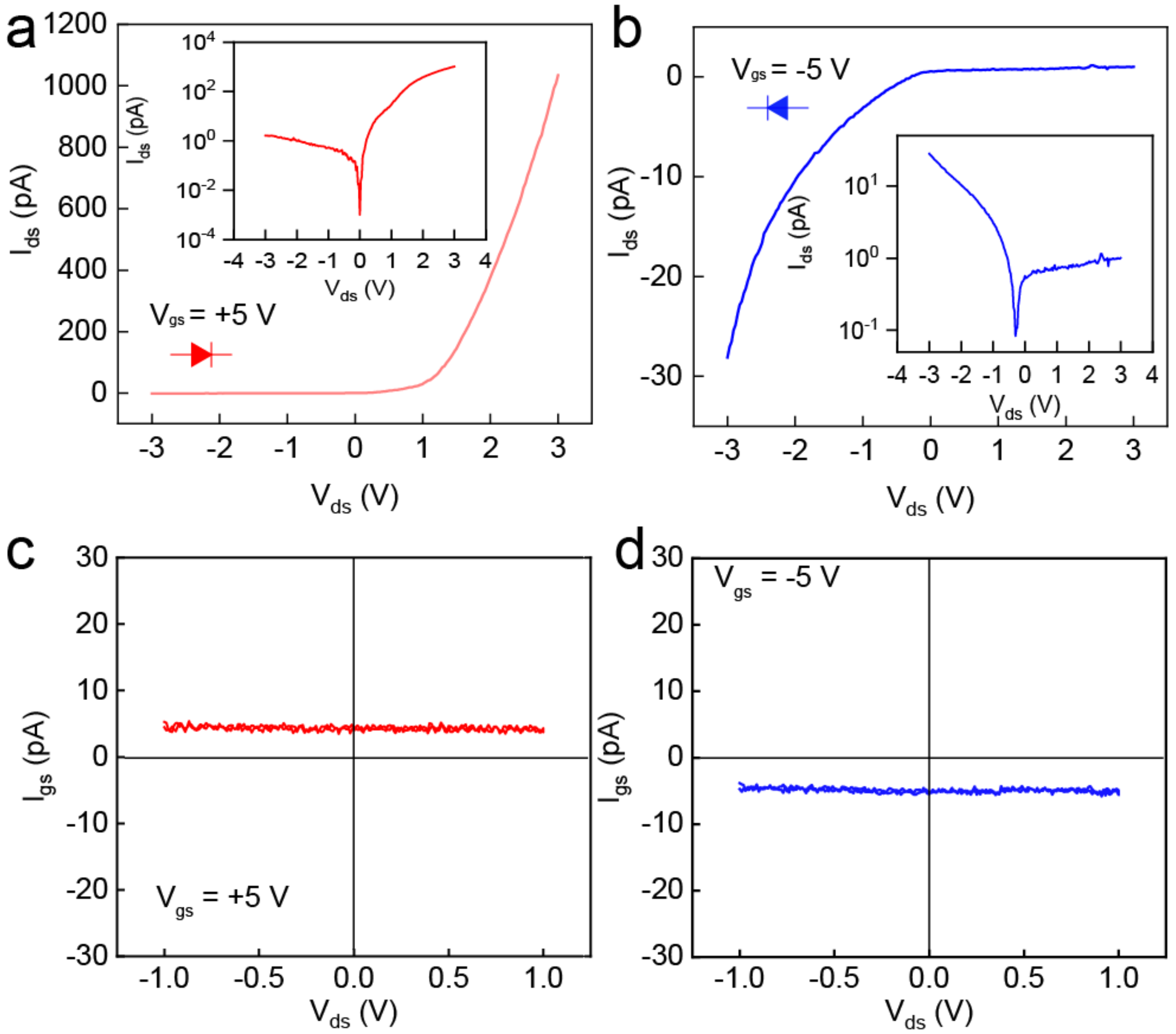

Figure S8 (a-b) Electrical properties of the InSe/GeSe vdWH-FET at different gate voltage. (c-d) the gateleakage current I_{leak} recorded corresponding to each I-V curve above.

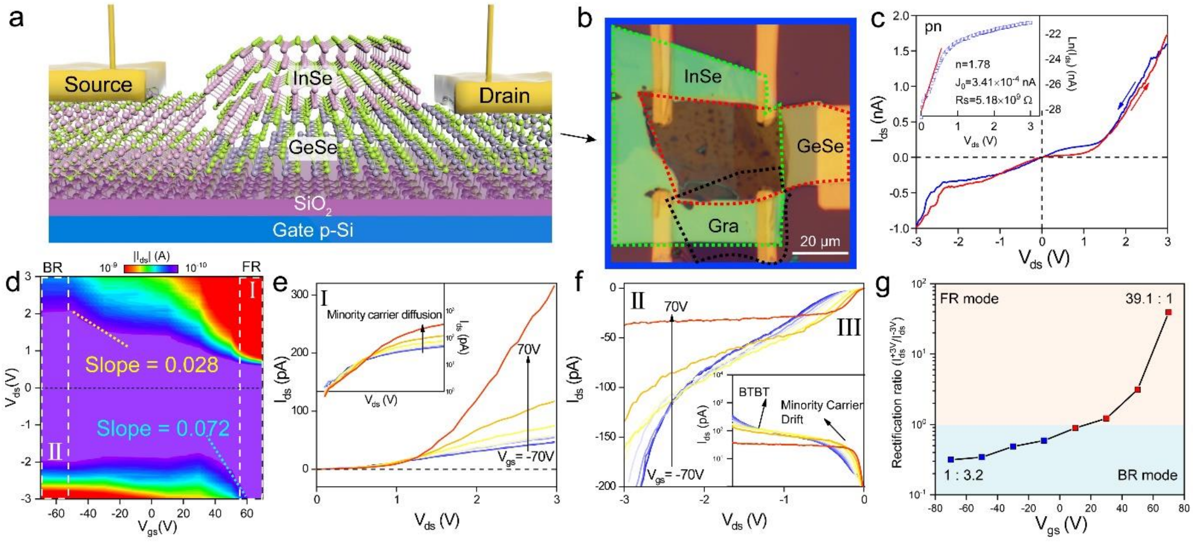

Figure S9 Schematic diagram (a) and optical microscope image (b) of a complete InSe/GeSe vdWH-FET device on SiO_2/Si substrate. (c) I_{ds}-V_{ds} characteristics of the InSe/GeSe device in linear and logarithmic (inset) scales. (d) Color map of output curves (I_{ds} vs V_{ds}) at different gate voltages. (e, f) Gate-tunable output curves ($I_{ds} - V_{ds}$) of the device under positive and negative drain bias in linear and logarithmic (inset) scales, respectively. (g) The rectification ratio of the vdW diode at different gate voltage.

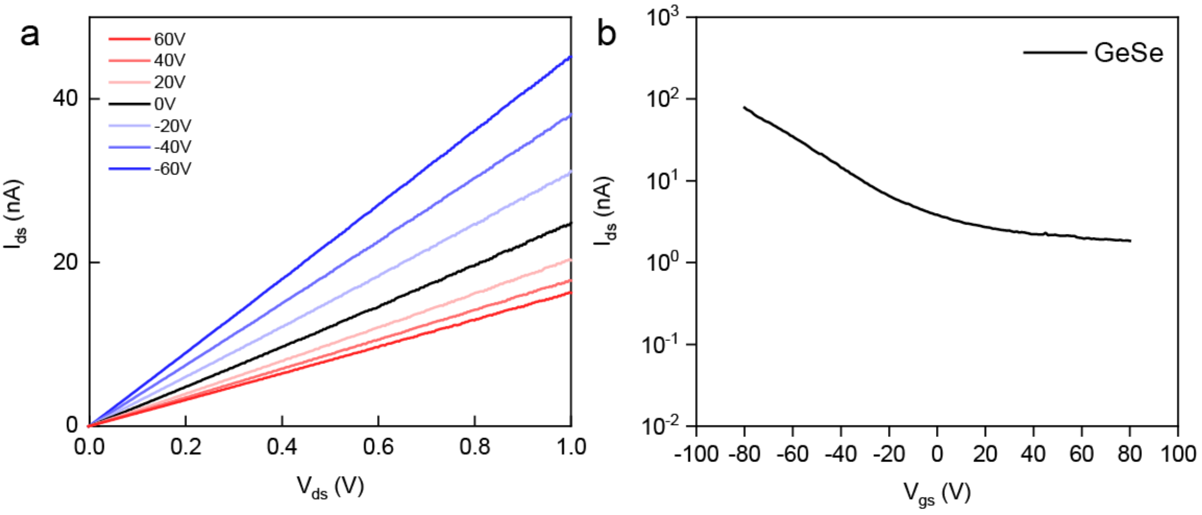

Figure S10 Output (a) and transfer (b) curves for Au/GeSe/Au devices.

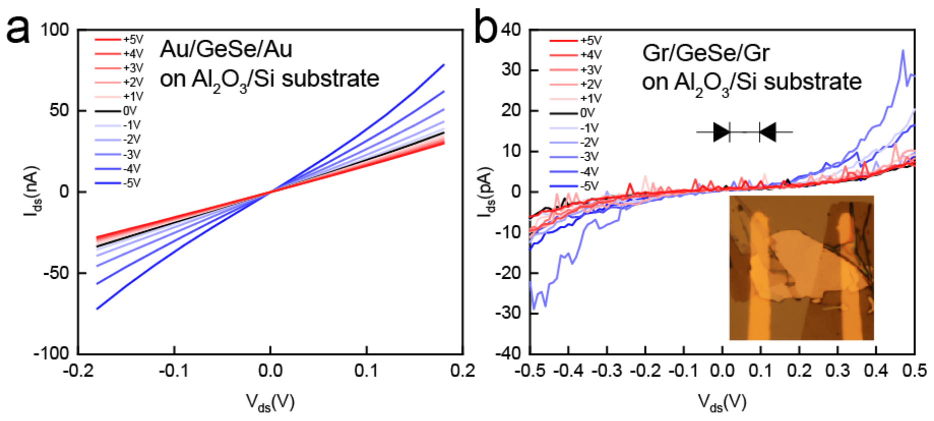

Figure S11 Output curves for Au/GeSe/Au (a) and Gr/GeSe/Gr (b) devices on Al_2O_3/Si substrate.

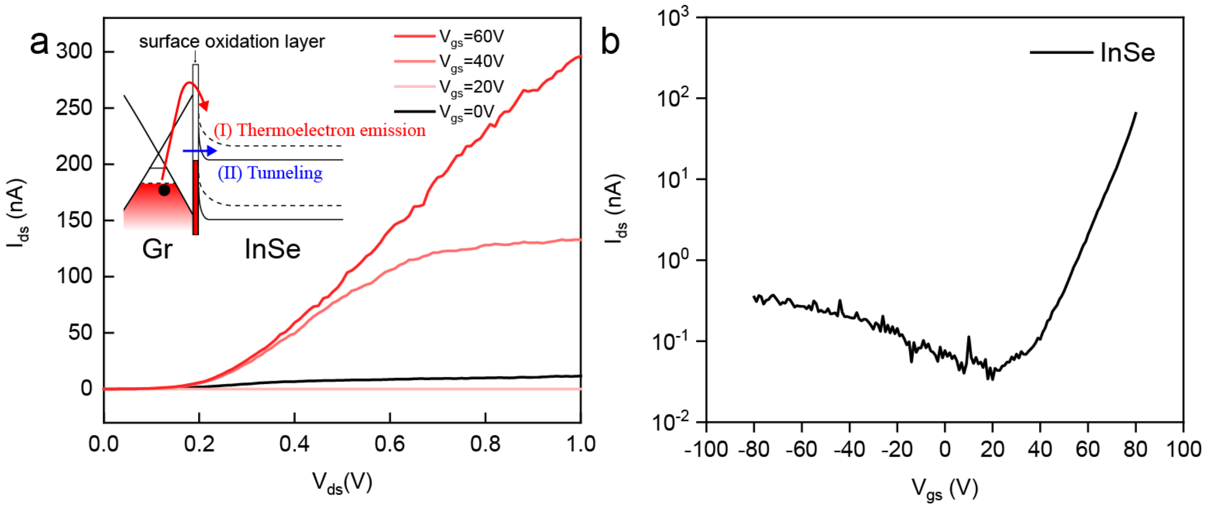

Figure S12 Output (a) and transfer (b) curves for the Gr-InSe-Gr device.

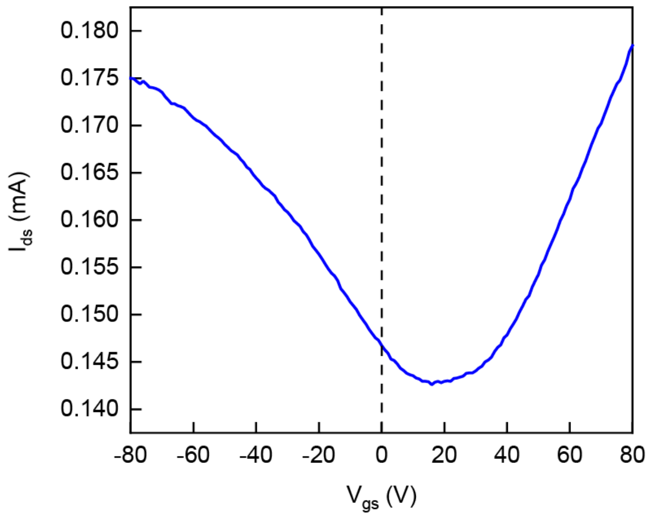

Figure S13 Transfer curve for the p-type graphene.

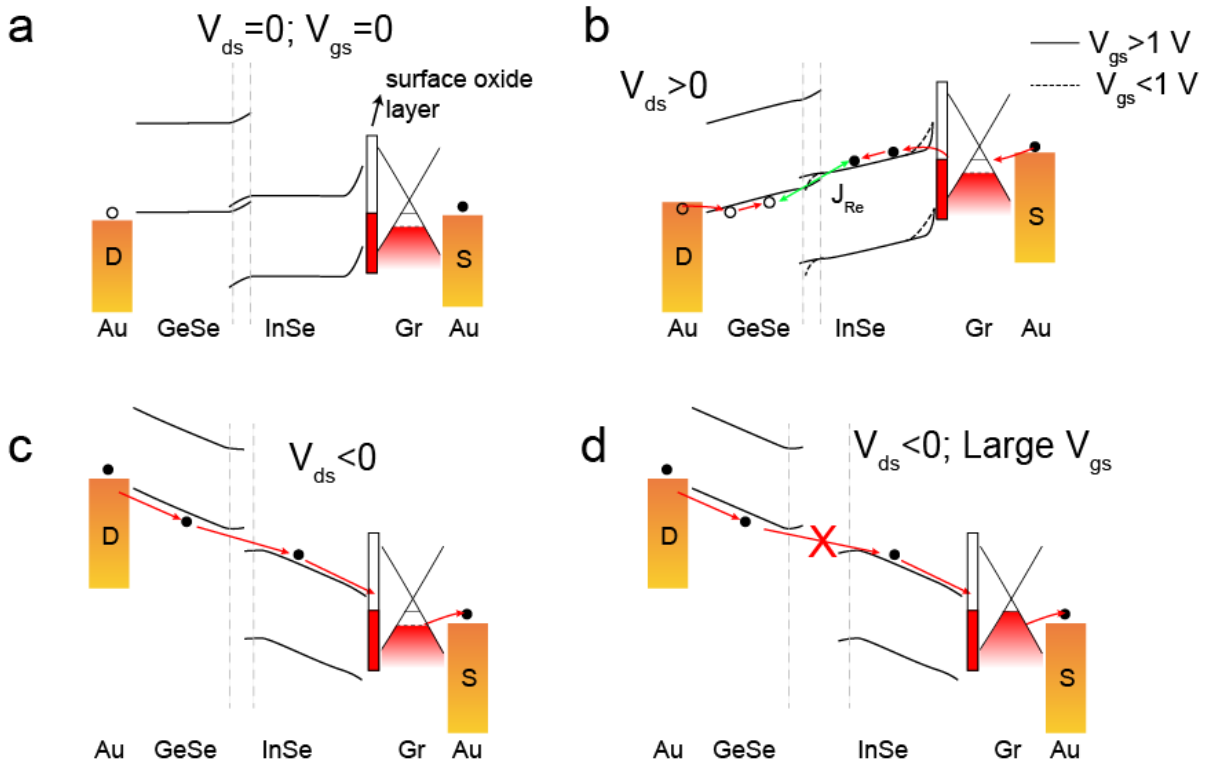

Figure S14 Band diagrams of the InSe/GeSe vdWH-FET at different bias condition.

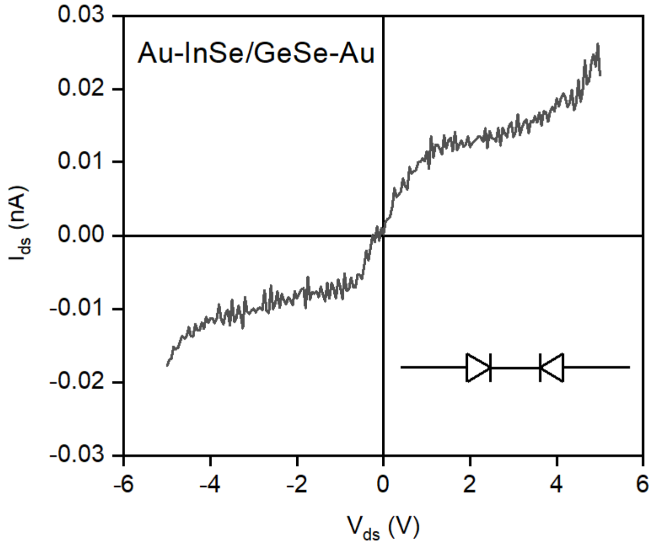

Figure S15 Current-voltage curve in dark for an Au-InSe/GeSe-Au device, which can be described as a pn junction sandwiched by a metal/semiconductor Schottky diode with opposite directions.

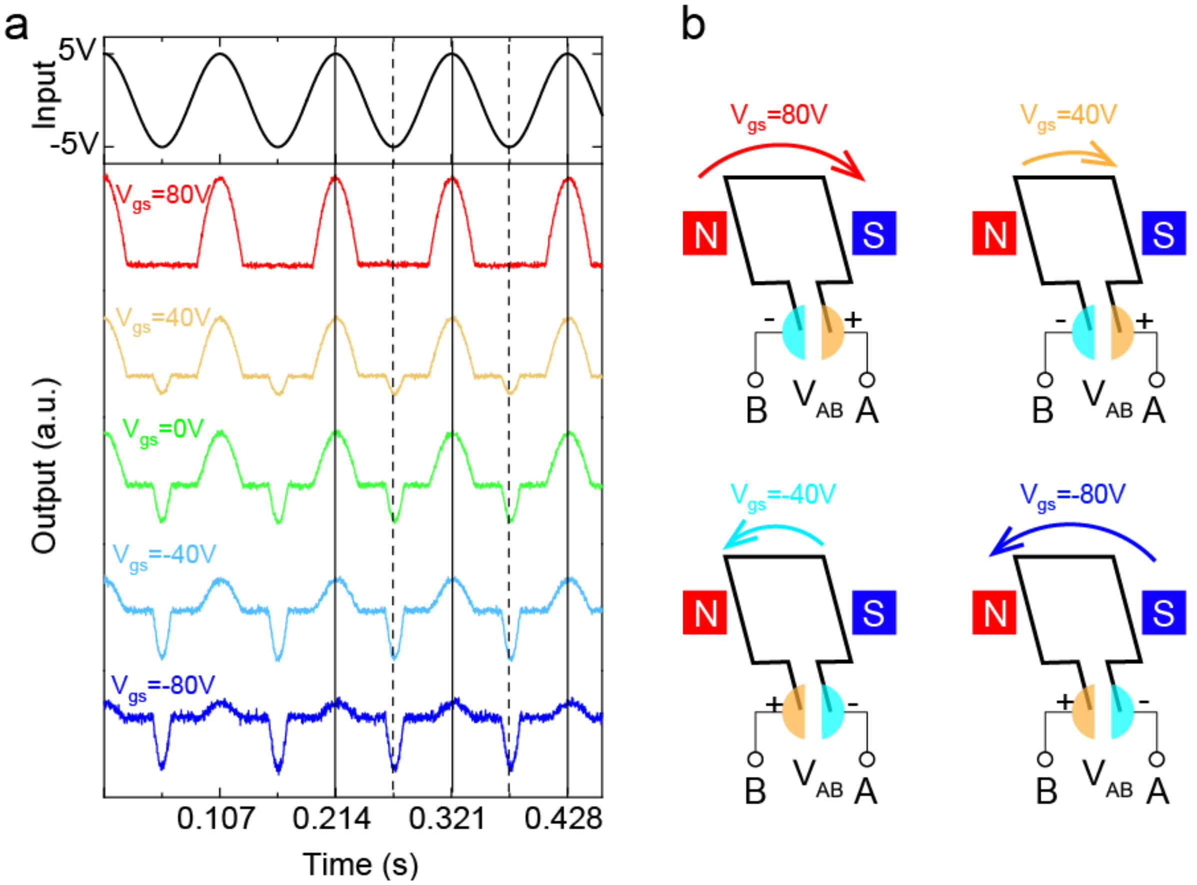

Figure S16 (a) The half-wave rectifying results of the InSe/GeSe vdWH on SiO_2/Si substrate under gate voltage of -80 to 80 V, respectively. (b) The proposed gate-controlled heterojunction bridge rectifier (GC-HBR) and its future application scenarios.

Note1. I-V characteristics of the InSe/GeSe vdWH-FET devices

We carried out current–voltage (I-V) measurements on the InSe/GeSe devices. Hysteresis induced by interfacial charge trapping/detrapping at the trap sites is also observed, but this could not affect the main result associated with this vdWH. In principle, at forward biases, the I-V curve of the pn junctions can be described by the well-known diode equation,

$$I = I_s \left\{ \exp\left[\frac{e(V_{ds} - IR_s)}{nkT} \right] - 1 \right\} \qquad (1)$$

where n is the diode factor, I_s is the saturation current, e is the electron charge, V_{ds} is the source drain voltage, k is the Boltzmann constant and T is the temperature, R_s is the series resistance.

Note2. The field effect mobilities of the InSe/GeSe vdWH-FET devices

The field effect mobilities were calculated by the relation,

$$\mu_{FE} = \left(\frac{dI_{ds}}{dV_{gs}}\right) \times (L/WC_iV_{ds}) \qquad (2)$$

where L and W are the length and width of the FET, C_i is the capacitance per unit area of the corresponding dielectric layer (2.1×10^{-3} F m^{-2} for 30 nm Al_2O_3 layer in these experiments). From the plots in Figure S7, our device shows electron and hole mobilities of 50.6 and 2.54 cm^2 V^{-1} s^{-1}, respectively. These values are comparable with the intrinsic InSe [5] (10-70 cm^2 V^{-1} s^{-1}) and GeSe [6] (0.8-6.0 cm^2 V^{-1} s^{-1}) under ambient condition without encapsulation by hBN or alundum.

References

[1] W. Li, X. Xiao, H. Xu, *ACS. Appl. Mater. Interfaces* **2019**, *11*, 30045.

[2] M. Huang, S. Li, Z. Zhang, X. Xiong, X. Li, Y. Wu, *Nat. Nanotechnol.* **2017**, *12*, 1148.

[3] W. J. Yu, Z. Li, H. Zhou, Y. Chen, Y. Wang, Y. Huang, X. Duan, *Nat. Mater.* **2012**, *12*, 246.

[4] J. Kang, D. Jariwala, C. R. Ryder, S. A. Wells, Y. Choi, E. Hwang, J. H. Cho, T. J. Marks, M. C. Hersam, *Nano Lett.* **2016**, *16*, 2580.

[5] W. Feng, X. Zhou, W. Q. Tian, W. Zheng, P. A. Hu, *Phys. Chem. Chem. Phys.* **2015**, *17*, 3653.

[6] W. C. Yap, Z. Yang, M. Mehboudi, J.-A. Yan, S. Barraza-Lopez, W. Zhu, *Nano Research* **2018**, *11*, 420.
